# Southern Medical Reports: Consisting of General and Special Reports of the Medical Topography, Meteorology and Prevalent Diseases in the Following States: Louisiana, Alabama, Mississippi North Carolina, South Carolina, Georgia, Florida, Arkansas, Tennessee and Texas; to Be Published Annually

**Published:** 1850-08

**Authors:** 


### BIBLIOGRAPHICAL NOTICES.

Southern Medical Reports: *Consisting of General and Special Reports of the Medical Topography, Meteorology and Prevalent Diseases in the following States: Louisiana, Alabama, Mississippi, North Carolina, South Carolina, Georgia, Florida, Arkansas, Tennessee and Texas; to be published annually.* Edited by E. D. Fenner, M. D., of New Orleans, Member of the American Medical Association, &c. &c.

(Concluded from page 422.)

In article twelfth we have an account of the New Orleans Charity Hospital. The extent of this hospital and its means of relief may be inferred from the fact, that, in 1849 the total admissions were 15,563; total discharges, 12,134. The deaths during the same time were 2,739. This shows a mortality of 17½ per cent. of the admissions—a large rate, but it occurred during the prevalence of one of the most destructive diseases—cholera. Many were admitted in a moribund state, or beyond the reach of remedies.

Of the above number of patients admitted, there were from

| | |
|---|---|
| United States, - - - - - | 1,782 |
| Foreign countries, - - - - | 13,034 |
| Unknown countries, - - - - | 142 |

The number of natives of Louisiana who were inmates of the hospital, was only 147. In how many ways is this great country the refuge of the people of the old world!

In another article, the organization of the State Medical Society of Louisiana is mentioned, and the nature and character of the standing committees are specified. They evince a laudable determination on the part of the physicians of Louisiana to keep up with the requirements of the present time, for the support and extension of the different branches of medical science.

All the reports hitherto noticed are from Louisiana.* Next follow those from Alabama. The initial one, by Dr. Bassett, on "the Climate and Diseases of Madison County," consists of much useful description, pointed and sometimes irrelevant references, and clinical reports. His biblical commentaries on the use of chloroform are at least amusing. When quoting the authors by whom cold bathing is recommended in Scarlatina, Dr. Bassett might have included Dr. John Bell, who, probably more than any other American physician, has emphatically exhibited its efficacy, first in his work on Baths and Mineral Waters, and subsequently in his published Lectures on the Theory and Practice of Physic, and in his late Treatise on Baths and the Watery Regimen.

The second article of the "Reports from Alabama," is entitled "Contributions to the Vital Statistics of Mobile, by George A. Ketchum, M. D." The mortality among the white population is much greater than among the black; and, in the former, more among the males than the females. The disparity in the case of the white males may be accounted for by their greater number, owing to so many of them coming without families to Mobile, to engage in business.

"Among the blacks the greatest mortality is among the infants under one year of age, and next between one year and ten."

Next comes an account of the Mobile Medical Society, and an

* The repetition before each article of the general heading, "Reports from Louisiana," is unnecessary. It would be enough were it to precede article first.

Abstract of its Proceedings. These consist of notes of interesting cases and remarks of the members, embodying, altogether, much useful information.

" Dr. Ketchum related the following instance of precocious development that he had met with in a family of negroes. The mother, Diana, was just thirteen years of age when her first child was born. This child, Tyra, was now twelve years and three months old, and has been menstruating eighteen months. She was three months advanced in pregnancy. Her breasts are large and full, though otherwise she has the appearance of a young girl of eight or nine years of age. Her younger sister, Mary, is just nine years of age, and has been menstruating regularly since the spring of 1848. If Tyra carries her fœtus until term, her mother will become a *grandmother* before she is twenty-six years of age."

Cases were related by Drs. Walkly, Ketchum, Ross, Anderson, and R. L. Fearn, the President, in which chloroform had been used with success. One was of " convulsions in a small child, in which all the usual remedies had failed to procure relief, and the child was fast sinking. He had chloroform administered by inhalation, and the convulsions had ceased, and the child had up to this time remained free from any recurrence of them." Dr. Walkly related another instance of the good effects of this treatment in the case of a child, fourteen months old, in whom the convulsions were confined principally to the right side. Dr. Anderson adduced a case coming under his own observation, confirmatory of this practice. The administration of the chloroform was kept up several hours, " on account of the tendency that the convulsions manifested to return. He thought that about ℥i. of the article had been used."

" Dr. Fearn reported two interesting cases of labor in which he had used chloroform with much benefit."

Dr. Ketchum has used chloroform successfully " in a violent case of hysterical convulsions occurring in a young woman, twenty-one years of age;" also " in a case of tedious and difficult labor." He found the topical application of chloroform to give relief in a very painful neuralgic affection of the face and one side of the scalp. "The patient was entirely relieved by wetting a handkerchief with a few drachms of the article, and applying it along the painful course of the nerves"—[the course of the pained nerves."] This article was used by Dr. Walkly in three cases of *trismus nascentium*, but without any good effect.

A case of nyctalopia was described by Dr. Ketchum, in which he gave a purgative of blue mass and rhubarb in the evening, and on the following morning fifteen grains of quinine were administered. " There was no return of the affection from this time."

The fourth article from the Alabama Reports, consists of Transactions of the State Medical Association, in the form of reports on Topography, Meteorology and the prevalent diseases. Dr. Fenner, after speaking of them in high terms, says: " They have been published in the New Orleans and Augusta Medical Journals, from which we shall select such as we think are most valuable."

An advantageous specimen of these reports is furnished in the paper by Dr. Bates, " On the prevailing diseases of a portion of Dallas County. Read before the Alabama State Medical Association, at its sitting in Wetumpka, on the 7th and 8th of March, 1849."

In the treatment of Bilious Remittent Fever, Dr. Bates rarely practices general bloodletting, unless in cases complicated with engorgement of internal organs—quite a common condition of things, he might have added—when local depletion is had recourse to. His course is a mildly antiphlogistic one. So soon as a remission is observed, he gives sulphate of quinine, and if there should still be some excitement, he combines with this article a small portion of ipecacuanha and morphia.

" Of the 16 cases of typhoid fever that came under my notice, 6 were whites and 10 were blacks ; all adults. Of the former, three proved fatal; of the latter, five. There were two cases occurring among the whites, and the same number among the slaves, that I term malignant typhoid, in contra-distinction to the others, from the severity of the symptoms and the rapidity of their course. The symptoms were different from those we usually see in typhoid fever, and in some respects assimilated those of malignant bilious fever. It is a difficult matter, I apprehend, to explain the combination of appearances, unless we suppose that the causes which produce remittent fever, modified considerably the idiosyncrasy of the individual in whom was developed the typhoid type."

Details of these cases are given by Dr. Bates.

Congestive Fever is next described by the author, who gives his views of its pathology, and the treatment which he adopts for its cure. There is no lack of active medication : " quinine and stimulants, with camph., opium and aromatics, are administered internally; while sinapisms, the hot air bath, dry frictions, and blisters to produce their full rubefacient effect, are applied to the extremities, spine and epigastrium. The patients bear quinine in large doses admirably, and I have frequently given 100 grains in a few hours without any other complaint than a little ringing in the ears. After reaction is in a measure effected, calomel in small doses, with opium, is given to correct the secretions, if necessary, while the quinine is continued to prevent a recurrence of the collapse. Perfect rest is enjoined the whole time."

In the treatment of the anginose variety of scarlatina, Dr. Bates recommends a mild antiphlogistic treatment—a mild laxative and a soothing diaphoretic, sometimes sponging with cold water. If the throat was greatly inflamed, "the tonsils were scarified with a common gum lancet, and then touched with a camel's hair brush and a solution of nit. silver, 10 grains per ounce, three times daily, until the soreness had in a measure disappeared. In some instances, the solution was gradually increased in strength from 22 to 25 grains. In not a solitary instance, where this course was pursued, was there ulceration or chronic engorgement, or enlargement of the tonsils, after the subsidence of the disease."

Georgia furnishes her contribution to these reports in a long and elaborately written paper by Dr. Pendleton, entitled "A General Report on the Topography, Climate and Diseases of Middle Georgia." In the positive, we are favored by the author with a description of the soil, face of the country and atmospherical states of the region. In the speculative and conjectural, he entertains us with his views of miasmata and the etiology of periodical fevers. The first is fresh and original; the second places him in the crowd of those who travel in the road of hypothesis.

The diseases of Middle Georgia have undergone, as we learn from Dr. Pendleton, a considerable change within the memory of man, both as regards their pathology and general fatality. "Formerly bilious remittent fever was a very fatal type of disease; now, I hesitate not in making the assertion, that uncomplicated remittent fever, as it prevails in Middle Georgia, never proves fatal, under a judicious and scientific treatment, if taken in time. I doubt not the virulence of the disease has greatly abated in late years, and even under the old plan of treatment, the mortality would not now be so great. But when we remember that the sole object of the practitioner of that day seemed to be to mercurialize his patient—particularly if one or two heavy charges of drastic purgatives did not succeed in ejecting the enemy, and during the whole course not a drop of cold water was ever allowed, no matter how dry the tongue or how burning the thirst, we wonder no longer at the greatness, but rather at the smallness of the mortality. Luckily for suffering humanity, a few wise heads soon discovered that every patient who obtained water by stealth, recovered, and those who did not, died, or suffered long before recovery; a consequent modification was made in the treatment, which has been still farther improved upon, under the benignant light of the Broussaian philosophy, until the monster has become a mere child, to be throttled and overcome by every tyro in medicine."

As regards the morbid effects of particular seasons on the animal economy, Dr. Pendleton, referring to the district described by him, tells us " what has grown to be an adage in the Southern States, that a wet spring and summer, with a dry fall, will produce a sickly season."

"Of what are generally termed idiopathic fevers, we have the common continued, inflammatory, bilious remittent and intermittent—the first two prevailing mostly in the cold months, and the last two in summer and autumn. The common continued fever frequently takes on a typhoid type after the first or second week, and hence has received that name by many physicians throughout the country. It is better known among the common people as the '*slow fever*,' from the tedious course it runs, frequently terminating either favorably or unfavorably at the end of the fourth or fifth week. I doubt not the true pathology of this disease is a sub-acute inflammation of the mucous membranes, originating in atmospheric vicissitudes, or supervening on the partial subduction of more violent fevers. The stimulating plan of treatment, as brandy, morphine and quinine, has consequently resulted most disastrously to all who have been brought under its influence. On the contrary, the *expectant* plan, of gentle antiphlogistics and counter-irritants, has relieved at least 19 cases in 20, as my tables will show."

Dr. Pendleton exhibits, in a tabular form, the number of cases of the different diseases which occurred in his practice in Hancock County, since 1843, and next of those of the deaths. We insert his summary view of the proportionate mortality. "Thus, out of

2,039, we have 60 deaths, being 2.94 per cent. The mortality of diseases of the digestive organs is 3.33 per cent.; the respiratory, 5.89; diseases peculiar to women, 3.2; brain and nervous system, 5.2; eruptive fevers, 4.4; idiopathic fevers, 0.34; and urinary, 1 in 52. It is remarkable that the mortality of idiopathic fevers is so small—there being of periodic fevers not a single death—of continued fevers, only 2 in 44; making a mortality of 4.4 per cent. This latter embraces that fearful type of fevers generally denominated typhoid. The result of the table certainly speaks volumes for the healthiness of our region in comparison with many other sections of the South."

Dr. Pendleton has written "On the Susceptibility of the Caucasian and African Races to the Different Classes of Disease." This paper first appeared in the Southern Medical Journal, and is now transferred to the "Southern Reports."

"The ratio of deaths, according to the number of cases, is 2.57 for the whites against 3.54 for the blacks." The whites are more subject to diseases of the primæ viæ than the blacks; the latter more to pulmonary affections than the former. There is greater call for medical assistance from the negro than from the white women, in the proportion of 15.2 to 10.5 per cent. The blacks are more subject to rheumatism, urinary affections and diseases of the teeth. The whites are much greater sufferers from idiopathic fevers, also, by a small per centage to diseases of the eye and exanthematous affections.

"With regard to the sexes, we find that the males are more subject to diseases of the digestive, respiratory, urinary and visual organs, as also the brain and nervous system, while the females, apart from diseases peculiar to them, are more liable to the exanthemata, rheumatism and diseases of the teeth. The females predominate over the males by a considerable per cent., in the general liability to disease. Thus, out of 1549 cases, we have 924 females, against 625 males. Abstract from those 204 for diseases peculiar to women, and they still have a considerable ascendency. But the diseases of females are less fatal in their character than those of males. Thus, out of the 924, we have 26 deaths, or 3.2 per cent. of females—while out of the 625, there are 20 deaths, or 3.2 per cent. of males."

"Perhaps the most remarkable fact connected with this table, as relates to the sexes, is the great preponderance of the males in idiopathic fevers. This being as 20.6 per cent. against 9.6; more than two to one."

Dr. Pendleton concludes his instructive paper in a tone of Christian philosophy, in the monition furnished to us by the infirmities of age and the more sudden inroads of violent disease:

"The lesson it teaches us of our mortality, is too obvious for the wise not to heed its healthful instruction and solemn warnings, and the good physician should always carry about him a medicine 'to minister to the mind diseased.' It is not found in our apothecaries' shops, nor is it indigenous to this clime; but still it may be obtained 'without money and without price.' It is the Elixir of Immortality."

"A Strange Case of Insanity," is the heading of a brief narrative of insanity, induced in a lady in Georgia, by fright from falling from a carriage. She was for some time in the Bloomingdale Asylum, but returned home without being cured of her malady. Her disposition had become, contrary to her wont, gay; and in conversation she evinced great sprightliness and wit. The house in the country in which she was residing took fire and was burned. But this disaster, by giving rise to terrible fright, "completely restored her to her right mind."

The organization and the names of the officers of the State Medical Society of Georgia, are furnished at page 344.

Under the head of Reports from Mississippi, we find an account of "the Topography, Meteorology and Climate of Jackson, the capital of Mississippi." By Dr. S. C. Farrar.

The author of this paper gives a lively sketch of the state of society and the causes of disease, and the defects of medical treatment, in the early settlement of Jackson, which, by the way, dates no farther back than 1832; and he contrasts them with the improvement in all these particulars at the present time. A large influx of both white and black settlers, the latter being slaves, unacclimated and over-tasked; the first owing to various speculations and anxious efforts to better their fortunes—the second to excessive field labor, furnished large materials for disease, the victims to which were increased by intemperance. "At almost every cross road a small cabin was erected, which, in common parlance, was denominated '*a dogery.*'"

Old physicians retired from practice; others abandoned it to engage in the less arduous and more attractive work of speculation. "Young physicians flocked to the State, chiefly from the schools of Lexington and Philadelphia, thoroughly indoctrinated with the peculiar opinions as to the origin and treatment of fever, entertained by two distinguished Professors filling the chairs of the practice of medicine in those schools; hence, in the treatment of fever, the lancet was frequently unsheathed, and calomel administered in large and repeated doses. When this failed to cure, ptyalism was resorted to; and that powerful anti-periodic, the Sampson of the materia medica in malarial fevers, quinine, was given in comparatively insignificant and feeble quantities. Up to this period, few if any had ventured to prescribe it in the heroic and jugulating doses of the present day."

The contrasted and gratifying picture to all this is given in the following terms by Dr. Farrar:

"Since then we have become better acquainted with the pathology and treatment of Southern diseases—we resort less to the lancet and heavy doses of calomel. We rely only on aperients, diaphoretics, opiates, salt water, enemata, cold drinks, sponging with cold water, affusions of cold water, sinapised foot baths, occasionally dry and wet cups and blisters; but above all, on the use of *quinine in sedative doses.* Since this change in practice, our intermittent and remittent fevers are more manageable, and even that terrible disease, *algid malignant intermittent* or congestive fever, has lost much of the horror it formerly inspired, and is far less intractable. But we are also exempt from many of the corroding cares and anxieties of 1833 and 1834, those years of speculation, when we were buoyed up one day with the expectation of riches by some lucky turn in the wheel of fortune, and the next, depressed by blighted hopes and ruined prospects. Now we enjoy more composure, we are better lodged and fed than formerly; our bodies are invigorated by labor and exercise; our supply of food is abundant, varied and wholesome, we are not constantly *upon the alert* for persons to victimise by bargain and trade; the days of banks and chimerical prospects have passed by, and now, *with few exceptions*, our citizens look not to lucky speculations nor to the placers of California, but to the bowels of our own soil, for gold. Most of the liquor-shops have been abandoned, demolished or converted to other purposes; and in every hamlet and town of the State, the Sons of Temperance have unfurled their banner, bearing on its ample folds the motto, '*love, purity and fidelity.*' They visit the habitation of the intemperate, carrying in their bosoms the feelings and sympathies of the good Samaritan, and on their tongues the language of love, and hope, and consolation. They admonish, they entreat the intemperate to abandon their habits, and flee the path that leads to ruin, disgrace, disease and death. Their friendly counsels make a deep impression, and frequently the inebriate is reclaimed, and goes forth again into society, with the sentiments, and aspirations, and dignity of man.

Now, acclimation, good food, pure water, exercise and temperance all contribute to render us less liable to disease; and when it comes, the system responds more readily to medicine."

Dr. Farrar describes an epidemic of measles which began in January, 1849, and prevailed for some weeks; and also one of erysipelatous fever, "the black tongue" of some writers, which appeared in February of the same year.

An overflow of the banks of Pearl river, on which the town of Jackson is situated, followed by a recession of the water, was productive of intermittent fever, from which scarcely a family escaped. Dr. Farrar, strengthening his remarks by the experience of Cleghorn, says, that "no disease is more disposed than tertian intermittents, to present anomalous symptoms, or to appear with a portion of the livery of other diseases." If it were necessary, this position might be still further strengthened by reference to Torti, Alibert, and others, who describe masked intermittents, (*febres larvatæ.*)

The second report from Mississippi is an article on Epidemic Cholera in the vicinity of Natchez. By C. H. Stone, M. D. This will repay perusal, but we have room for only one extract:

"A comparison of the different reports will no doubt show *the almost simultaneous formation of the cholera poison throughout a great, if not the whole, extent of the valley of the lower Mississippi;* not travelling *up* the river from New Orleans, nor *down* from Memphis or Vicksburg, but like a vast, dread pall, impending over this great valley, and settling here and there, first on its heart and great trunk, then its numerous rivers, lakes, and extensive plains, shrouding thousands in death."

Following this paper is another on Epidemic Cholera and its Preventive Treatment. By G. S. Magoun, M. D. It has the merit of brevity. He designates it as "a crude essay." He is either too modest in thus underrating the value of his production, or he is too frank in telling his readers what little pains he has thought necessary to take for their edification.

Dr. Clemens, of Macon, Mississippi, relates an operation for the removal of one half of the inferior maxilla, for osteo-sarcoma. The subject was a negro, aged about thirty-seven years. Dr. C. first saw him in June, 1847, and learned that the disease had begun to show itself in February, 1845. The use of iodine was persevered in for nearly a year, but without any good effect. The general health was much improved under the use of tonics, prescribed by Dr. C. On the 12th of September, 1847,

Dr. Clemens performed the operation of removal of the diseased bone "by sawing through just beyond the angle on one side, and near the symphysis of the chin on the other." On October 14th the wound had entirely healed, and the patient was discharged, apparently quite well. This is the first chapter in a surgical operation beyond which the narrator sometimes forgets to publish. The second, far from being an uncommon one, tells a different story, viz. of renewed disease, declining health, and finally death. Dr. Clemens, with becoming honesty of purpose, continues the history of this case, by telling us of his having, in April, 1849, seen the patient, for such he had then become, who complained of severe pain in the ramus of the maxilla on the side from which the tumor had been extracted. The advice of Dr. C. to have the affected bone removed at the articulation, was neglected The disease continued to make rapid progress—a tumor larger than a man's fist had grown out, and the integuments at the lower part were ulcerated, and a dark fungus protruded. This was the state of things in August, when Dr. Clemens performed another operation, after having placed the patient under the influence of chloroform, and the carotid artery tied, as a precautionary measure, at the point where it is crossed by the omo-hyoid muscle. The operation is thus described :

"I now made an incision, commencing over the articulation and carried downwards along the posterior margin of the tumor, terminating at a point about midway between the angle and symphysis. Another incision was then made, commencing just below the first, and carried along in front of it, (so as to include the cicatrix of the former operation,) and terminating with it. The tumor was now separated, as far as practicable, from its attachments, the capsular ligament of the joint divided—the tumor turned over from behind, forward, pressed downward, the temporal muscle divided at its insertion, and the removal was effected.

"The hæmorrhage was very trifling. The cavity was lightly filled with lint, and the edges of the wound brought in apposition with sutures and adhesive strips, and the parts supported with a light bandage. The healing process went on rather slowly, though by the fortieth day the wound was healed throughout. The ligature around the carotid came away on the twenty-fifth day. On the forty-second day from the operation the patient was again discharged, apparently quite well."

Notwithstanding these flattering appearances, in the course of three months the upper jaw on the same side became the seat of disease, manifested by fever, and a tumor nearly the size of a hen's egg. The general health had again become bad. Under all the circumstances of the case, Dr. Clemens wisely declined any farther operation for the relief of the patient.

Dr. Joseph J. Pugh communicates a brief account of the "Artesian Springs" in Madison county, Mississippi. The water is *acidulated* chalybeate. Dr. Pugh bears testimony to its beneficial operation in various diseases—debility of the digestive organs, including diarrhœa and dysentery, also menorrhagia, amenorrhæa, fluor albus, functional diseases of the kidneys, unattended by inflammation; also in cutaneous affections, and in the distressing gastric irritation consequent on uterine diseases.

The first and only report from Tennessee is "On the Commencement, Prevalence, Fatality, Treatment, &c., of Pestilential Cholera, in Memphis and its vicinity; with the prominent facts bearing upon the unsettled question of its imported or domestic origin. By Lewis Shanks, M. D., of Memphis, Tenn."

Dr. Shanks advocates the contagious nature of cholera, basing his opinion on the facts observed by him and others, respecting the diffusion of the disease along the Mississippi and its tributaries. He writes:

"The question might fairly be asked upon this statement of facts —If cholera was not imported here from New Orleans—if it originated here from epidemic influence—why should nearly all the cases, for the first fifteen or twenty days, have occurred on a string of landing some two miles in length, contiguous to the steamboat channel, and no cases occur in the town, only a few hundred yards distant from the landing, but the two under the circumstances specified?"

From South Carolina, Dr. Thomas Y. Simons, of Charleston, sends a Report, which, by the way, first appeared in the pages of our able contemporary, the *Charleston Medical Journal and Review*, for September, 1849. It is entitled "Observations on the fever which is developed in the city of Charleston after exposure to the country air, during the summer and autumn, and which is hence called Country Fever."

The treatment of this fever is briefly sketched by Dr. Simons as follows:

"My plan is, if the bowels are confined to give a good mercurial purge of rhubarb and calomel, and upon the remission of the fever to give two grains of quinine every two hours, until a sense of ringing in the ears or partial deafness ensues, or until the exacerbation supervenes—keeping the liver secreting by the use of blue pill every four hours during the day, and the bowels relieved when necessary, by injections; avoiding active cathartics, if possible. This plan is continued until the fever is arrested, which I have found generally to be after the second or third invasion. Sinapisms, blisters and rubefacients, or cold evaporating applications, are used likewise when indicated. I should not hesitate to use bleeding generally and topically during the exacerbation, but I deem these should be done in the earlier stage of the disease."

The author offers some judicious remarks of a prophylactic nature, and concludes his paper by the following suggestion.

" It is, to ascertain the localities which are healthy and those which are liable to fevers—the means of preserving the health of those which are now healthy and of correcting and ameliorating those which are sickly. It is a noble and philanthropic object, and one which should bring forth the energies and observation of every intelligent physician in the State. The State Medical Association has appointed a committee as regards this subject, which committee, it is to be hoped, will receive ample materials of information in the different districts and parishes in the State."

We can only state the fact of the appearance in these Reports, of an instructive paper on the Yellow Fever of Charleston, S. C., in 1849, by the editor of the Charleston Journal, in which it originally appeared.

Dr. Holmes, of Maybinton, S. C., gives his experience of the good effects of nitrate of silver, especially applied in anginose affections; of strychnine in paralysis; and belladonna in pertussis.

The next article is a brief notice of the organization and proceedings of the South Carolina Medical Association.

We may anticipate the collection and publication, in a few years, of an immense body of invaluable matter on the Medical Topography and Climate of the different sections of nearly every state in the Union, obtained through the methodical observations made by their Medical Societies.

Texas sends a report from Dr. Wright, surgeon in the United States Army, on " the Topography of San Antonio, and the epidemic cholera that prevailed there in the spring of 1849."

Dr. Wright begins his paper by appositely remarking: " He who would sit down to write a paper on Cholera Asphyxia, at the present day, finds himself in many essential respects, like him, who would indite a treatise on variola, or pertussis, or intermittent fever; or like him who is constrained to elaborate a Fourth of July oration, or a eulogy on General Washington." Still is Dr. Wright, writing from personal observation, worthy of being read.

Dr. Jarvis, surgeon United States army, sends his quota, in the shape of a "Report on the rise, progress and decline of epidemic Cholera in the valley of the Rio Grande." The remarks just made regarding Dr. Wright, applies to the author of this paper.

The portion of the volume before us, called Excerpta and Miscellanies, consists of 1. "Experimental Researches on the action of quinine, especially in large doses. A memoir submitted to the Academy of Science.—By M. Brecquet. Report of MM. Andral, Rayer, and Lallemand. 2. On the treatment of the West India remittents and intermittents by quinine. By Dr. D. Blair, of Demarara. 3. Does calomel really expel the biliary secretion? By Dr. Michea. 4. The law relating to the importation of adulterated drugs, medicines, and chemical preparations into the United States."

It is very justly remarked in the opening sentence of article 4th, "The medical profession, as well as the entire community throughout the Union, are greatly indebted to Dr. T. O. Edwards, late member of Congress from Ohio, for his indefatigable and finally successful efforts to procure the passage of this most salutary and important law." Our readers will see, by reference to our advertising sheet, that Dr. Edwards has returned to the ranks of the profession, and will bring his talents and attainments into the service of the Medical College of Ohio, in which school he has been appointed Professor of Materia Medica and Pharmacy.

Notices of the medical colleges of the south and south-west, and of American medical journals, conclude the volume of "Southern Reports."

The pains which we have taken to exhibit to our readers the chief features, and most interesting details of the first volume of Dr. Fenner's *Southern Reports*, are the best proof of our desire to see a second volume next year. It would be a source of pleasing reflection to us if we could believe, that the language of praise and occasional criticisms which we have uttered, will encourage him in his future endeavours, and furnish him with hints to make his subsequent course more easy.

